# COMP report: CPQR technical quality control guidelines for brachytherapy remote afterloaders

**DOI:** 10.1002/acm2.12272

**Published:** 2018-02-07

**Authors:** Normand Frenière

**Affiliations:** ^1^ Département de Radio‐Oncologie Centre intégré universitaire de santé et de services sociaux de la Mauricie‐et‐du‐Centre‐du‐Québec Centre hospitalier affilié universitaire régional Trois‐Rivières Québec G8Z 3R9 Canada

**Keywords:** brachytherapy remote afterloaders, quality control guidelines, radiation treatment therapy equipment

## Abstract

The Canadian Organization of Medical Physicists (COMP), in close partnership with the Canadian Partnership for Quality Radiotherapy (CPQR) has developed a series of Technical Quality Control (TQC) guidelines for radiation treatment equipment. These guidelines outline the performance objectives that equipment should meet in order to ensure an acceptable level of radiation treatment quality. The TQC guidelines have been rigorously reviewed and field tested in a variety of Canadian radiation treatment facilities. The development process enables rapid review and update to keep the guidelines current with changes in technology (the most updated version of this guideline can be found on the CPQR website). This particular TQC details recommended quality control testing of brachytherapy remote afterloaders.

## 1 INTRODUCTION

The Canadian Partnership for Quality Radiotherapy (CPQR) is an alliance amongst the three key national professional organizations involved in the delivery of radiation treatment in Canada: the Canadian Association of Radiation Oncology (CARO), the Canadian Organization of Medical Physicists (COMP), and the Canadian Association of Medical Radiation Technologists (CAMRT). Financial and strategic backing is provided by the federal government through the Canadian Partnership Against Cancer (CPAC), a national resource for advancing cancer prevention and treatment. The mandate of the CPQR is to support the universal availability of high quality and safe radiotherapy for all Canadians through system performance improvement and the development of consensus‐based guidelines and indicators to aid in radiation treatment program development and evaluation.

This document contains detailed performance objectives and safety criteria for *Brachytherapy Remote Afterloaders*. Please refer to the overarching document *Technical Quality Control Guidelines for Canadian Radiation Treatment Centres* 1 for a programmatic overview of technical quality control, and a description of how the performance objectives and criteria listed in this document should be interpreted. The development of the individual TQC guidelines is spearheaded by expert reviewers and involves broad stakeholder input from the medical physics and radiation oncology community.^2^

All information contained in this document is intended to be used at the discretion of each individual center to help guide quality and safety program improvement. There are no legal standards supporting this document; specific federal or provincial regulations and licence conditions take precedence over the content of this document.

## 2 SYSTEM DESCRIPTION

Brachytherapy is the placement of encapsulated radionuclides or a miniaturized x‐ray tube in, or adjacent to, tissue which has been prescribed a radiation dose.^3^, ^4^, ^5^, ^6^, ^7^, ^8^, ^9^, ^10^ This practice offers unique advantages to the management of several treatment sites and has been used to complement or replace external beam radiation therapy since the onset of radiation oncology.

Remote afterloading equipment was developed to reduce, and in many cases eliminate, the radiation exposure to members of the staff. With remote afterloading systems the user does not directly handle the radioactive source and the patient is irradiated in a shielded room with staff operating and monitoring the process remotely.

High dose rate (HDR) refers to treatment dose rates larger than 20 cGy/min. For all HDR remote afterloaders, a single and small (< 1 mm × 5 mm) radioactive source (mostly iridium‐192, rarely cobalt60), laser‐welded to a metallic cable, is moved out of the safe by a motor‐drive mechanism to step along the prescribed positions (dwell positions) with different irradiation times (dwell times). The user can preselect dwell positions and dwell times at selected positions in a number of applicator lines. The remote afterloader could receive up to two such sources with two independent cables permitting dose delivery in two applicator lines simultaneously. The source strength is approximately 40,000 cGy cm² h^−1^ (activity of ~370 GBq) on installation of a new iridium‐192 source, while it is of 23,000 cGy cm² h^−1^ (activity of ~74 GBq) for a new cobalt‐60 source. Because iridium‐192 has a relatively short half‐life (73.8 days), the sources are usually replaced about every 3 months. Cobalt‐60 has a longer half‐life (1 925 days or 5.27 yr), offering less frequent source replacement to every 5 yr. Typical HDR irradiation times are 5 to 30 min, and a treatment course may consist of several fractions.

Another form of treatment is pulsed dose rate (PDR) treatment. With a PDR device, irradiations are given in short “pulses” with the total treatment being given in 48 to 72 h. The mechanism for PDR units is very similar to that used in HDR units. PDR remote afterloaders also use a single iridium‐192 source attached to a cable. However, the source strength for these units is typically only 10% of the source strength of an iridium‐192 HDR unit.

High‐dose rate miniaturized x‐ray tube remote devices, along with intravascular and cardiovascular brachytherapy remote afterloaders using beta emitting radioactive sources, are beyond the scope of this document.

Various recommendations for brachytherapy quality assurance have been reported in the literature.^11^, ^12^, ^13^, ^14^, ^15^, ^16^, ^17^, ^18^ “Per treatment” tests must be executed prior to each treatment. “Treatment day” tests must be scheduled before treating the first patient of the day. For PDR remote afterloaders where treatments may last several days, “treatment day” tests should be performed prior to the initiation of the treatment.

**Table 1:** Per treatment quality control tests

Designator	Test	Performance
		Action
Per treatment (executed prior to each treatment)		
T1	Plan data transfer from treatment planning computer	Data integrity
T2	Plan dwell times adjustment	See note
T3	Minimum dwell times	See note
T4	Plan catheters’ connection to remote afterloader	Reproducible
T5	Complete source retraction	Functional
Treatment day (or per treatment for PDR)		
D1	Treatment interrupt	Functional
D2	Console displays (treatment status indicator) and key switch	Functional
D3	Date, time, and source strength in treatment unit	See note
D4	Source (and dummy) positional accuracy	2 mm
D5	Dwell time accuracy	2%

**Table 2:** Quarterly quality control tests

Designator	Test	Performance
		Action
Quarterly (or at source replacement)		
Q1	Mechanical integrity of applicators, guide tubes, connectors	Functional
Q2	Internal battery power supply (power failure recovery)	Functional
Q3	Source/dummy interlocks	Functional
Q4	Dummy wire positional accuracy	3 mm (1 mm see note)
Q5	Radiological source positional accuracy	1 mm
Q6	Source strength calibration	5%
Q7	Source homogeneity	Reproducible
Q8	Records	Complete

**Table 3:** Annual quality control tests

Designator	Test	Performance
		Action
Annually		
A1	Hand crank operation	Functional
A2	Leakage radiation	Reproducible
A3	Multi‐channel indexer function	Functional
A4	Dwell time accuracy	1%
A5	Timer linearity	1%
A6	Transit time/transit dose reproducibility	Reproducible
A7	Dosimetric length of applicators and guide tubes	1 mm
A8	Applicators and templates dimensions	Reproducible
A9	Shield integrity of shielded applicators	Reproducible
A10	X ray marker positional accuracy	1 mm
A11	Document staff review of emergency response procedures	Complete
A12	Independent quality control review	Complete
A13	PDR sequencing (for PDR only)	Functional

## 3 RELATED TECHNICAL QUALITY CONTROL GUIDELINES

In order to comprehensively assess brachytherapy remote afterloader performance, additional guideline tests, as outlined in related CPQR Technical Quality Control (TQC) guidelines, must also be completed and documented, as applicable. Related TQC guidelines, available at http://www.cpqr.ca/programs/technical-quality-control/, include:

- Safety Systems^19^
- Major Dosimetry Equipment^20^
- Treatment Planning Systems^21^

## 4 TEST TABLES

### 4.A Notes on per treatment tests

**Table:** 

T1	Plan data imported from a treatment planning system into the treatment console should be verified for source strength, dwell positions, and dwell times. In case of many dwell positions, the verification of a sub‐set of positions is acceptable. For a plan already present in the treatment console, the same verification should be made to assure proper plan selection
T2	Plan dwell times adjustment by the treatment console for the treatment date should be verified (by an independent calculation: hand calculation, decay factor chart, or software calculation). Action level will depend on the treatment console decay frequency and time resolution; express in percentage of difference and/or in second
T3	Minimum dwell time should be verified against the device driven limits. Those limits should take into account both the effect of transit dose and positioning reproducibility. Some remote afterloader systems might have a positioning reproducibility dependence on dwell time. All dwell times should be equal or greater than set limit
T4	Catheters/applicators connections to remote afterloader indexer channels must match plan
T5	Survey the treatment room and patient to ensure that source has been completely retracted

### 4.B. Notes on treatment day (or per treatment for PDR) tests

**Table:** 

D1	During source exposure, verify that the non‐emergency interrupt button (if equipped) retracts the source to its safe and shielded position
D2	On the treatment console, displays should be verified. At minimum, treatment status indicators should be verified by exposing a source. Indicators could be visual and audible. When a treatment key is available, its deactivation should prevent source exposition
D3	Remote afterloader console date and time are properly set. Decayed source strength is accurate compared to an independent calculation (hand calculation, decay factor chart or software calculation), taking into account treatment console decay frequency
D4	Verify accuracy of source drive mechanism positioning. A visual inspection with a camera is acceptable. Apply also to dummy drive mechanism if used to measure catheter length
D5	Comparison of dwell time accuracy with external standard such as a stopwatch. The dwell time used should be sufficiently long such that errors in the measurement of the time (e.g., reaction time of the observer) are less than 1%

### 4.C Notes on quarterly tests

**Table:** 

Q1	Verify the applicators, guide tubes, and connectors are exempt of damage (excessive wear, kinks, etc.)
Q2	The configuration of this test will depend on the design of the facility and equipment. Safety is the concern and tests should be designed accordingly. The first objective is to verify that the equipment safely retracts the source wire after a power failure. The second objective is to verify that the equipment properly records treatment delivered before power failure and permits to resume the treatment after power recovery
Q3	Verify functionality of remote afterloader interlocks related to source and dummy wires. This includes incorrect connection of applicator to transfer guide tube, incorrect connection of transfer guide tube to remote afterloader, and obstruction
Q4	Verify accuracy of dummy drive mechanism positioning. The purpose is to assure proper obstruction detection by assuring no false positive or false negative obstruction. If dummy drive mechanism is used to measure catheter length for treatment planning, then the action level should be lowered to 1 mm
Q5	Accuracy of source drive mechanism to be verified. Autoradiographs or ion‐chamber measurements could be used. If visual checks with in‐room cameras are to be used, source positioning in the cable construction should be verified independently
Q6	Comparison of measured source strength with manufacturer supplied value. On installation of a new source, source strength must be measured using calibrated re–entrant chamber and electrometer traceable to a national standards laboratory. The re‐entrant chamber and electrometer should have been calibrated within the last 2 yr. Measured source strength should be used for planning and treatment purposes. Discrepancies greater than 5% between the measured and the manufacturer's supplied source strengths must be investigated. This action level could be lowered to 3%^22^ if the manufacturer supplied source strength offers such precision. Stability of re‐entrant chamber should be verified prior to use. A second qualified medical physicist should perform a check of the calibration
Q7	Visual check on film that the radioactive material is evenly distributed in the encapsulated source. Most important for sources composed of multiple source pellets
Q8	Documentation relating to the daily quality control checks, preventive maintenance, service calls, and subsequent checks must be complete, legible, and the operator identified

### 4.D Notes on annual tests

**Table:** 

A1	Manual emergency hand crank functionality should be verified with manufacturer service engineer. It is desirable that each person responsible to operate the hand crank, in an emergency situation, practices its operation annually when a dummy wire replaces the source wire
A2	Monitor leakage radiation to check afterloader's safe integrity. Intensity of leakage radiation must be lower than the value set by manufacturer and local regulations
A3	Verify multichannel indexer functions properly. The wire must be sent to the proper programmed channel
A4	Comparison of dwell time accuracy with an external standard performed more rigorously than the treatment day test
A5	Verification of the linearity of the timer over a clinically relevant range. The action level represents deviations of measured values from those calculated using a linear fit to the measured data
A6	Reproducibility of transit time effect or transit dose effect or source speed between dwell positions. Can be verified using autoradiographs, ion‐chamber measurements, or visual checks with in‐room cameras. A fixed and reproducible applicator geometry is required to assure the same wire drive speed
A7	Reusable applicators and transfer guide tubes length should be measured to verify dosimetric lengths if used clinically as nominal values. This measurement could also verify that no debris has come into the lumen
A8	Verify physical dimensions of reusable applicators and templates (e.g., diameters, angles, shields). They must match dimensions used in the planning process
A9	Verify shielding integrity of shielded applicators. Visual and radiographic inspections should be performed
A10	Check x‐ray markers positional accuracy if used clinically for source positioning. If markers are only used to draw the applicator path, then only the integrity should be checked
A11	The configuration of this test will depend on the design of the facility and equipment, and local regulations. All staff should review the emergency procedures when a source fails to retract properly and remains outside the safe
A12	To ensure redundancy and adequate monitoring, a second qualified medical physicist must independently verify the implementation, analysis, and interpretation of the quality control tests at least annually
A13	For PDR only, verify pulse sequencing functionality according to manufacturer's recommendations

## CONFLICT OF INTEREST

No conflicts of interest.

## DISCLAIMER

All information contained in this document is intended to be used at the discretion of each individual centre to help guide quality and safety program improvement. There are no legal standards supporting this document; specific federal or provincial regulations and licence conditions take precedence over the content of this document. As a living document, the information contained within this document is subject to change at any time without notice. In no event shall the Canadian Partnership for Quality Radiotherapy (CPQR) or its partner associations, the Canadian Association of Radiation Oncology (CARO), the Canadian Organization of Medical Physicists (COMP), and the Canadian Association of Medical Radiation Technologists (CAMRT), be liable for any damages, losses, expenses, or costs whatsoever arising in connection with the use of this document.
